# Epidemiologic and Clinical Divergence of MRSA USA100 and USA300 in the United States

**DOI:** 10.3390/antibiotics15040372

**Published:** 2026-04-04

**Authors:** Camille André, Michael S. Gilmore

**Affiliations:** 1 Department of Ophthalmology, Massachusetts Eye and Ear, Harvard Medical School, Boston, MA 02114, USA; candre2@meei.harvard.edu; 2 Infectious Disease Institute, Massachusetts Eye and Ear, Harvard Medical School, Boston, MA 02114, USA; 3 Department of Microbiology and Immunobiology, Harvard Medical School, Boston, MA 02115, USA

**Keywords:** MRSA, USA300, USA100, hospital-associated, community-associated, virulence, antibiotic resistance

## Abstract

Methicillin-resistant *S. aureus* (MRSA) is listed by the World Health Organization as a priority pathogen posing a major worldwide threat to public health. Two lineages of MRSA predominate as causes of human infections in the U.S.: USA300 and USA100. Although they are most often grouped together as MRSA, these two lineages differ in pathogenetic mechanisms in important ways. The epidemic spread of these two dominant lineages has been problematic because of the multidrug-resistant profile of USA100 and the virulence of USA300, as well as their ability to adapt to both community and hospital environments. In this perspective, we examine what is currently known about their distinctive biology and the consequent differences in infections caused by these two main MRSA epidemic clones. The purpose of this perspective is to provide critical insights to the clinical microbiology community to stimulate further research to inform the design of new prevention and management strategies for MRSA.

## 1. Introduction

*Staphylococcus aureus* is a well-known colonizer of the skin, respiratory tract, and other mucosal surfaces of healthy individuals [1], and partly as a result of this, it is also a leading cause of a range of community-acquired and nosocomial infections worldwide [2]. Because of the added dimension of antibiotic resistance, methicillin-resistant *S. aureus* (MRSA) is listed by the World Health Organization as a priority pathogen posing a major threat to public health globally [3].

After being largely confined to the hospital environment for nearly 30 years, specific lineages of MRSA became established in the community in the 1990s [4] and have now become leading causes of antibiotic-resistant infections in both settings [5]. Despite a wide range of diversity within the species, and the existence of methicillin-resistant strains for over 50 years, only a few discrete MRSA lineages predominate. This raises fundamental questions: Why do certain clones prevail? What biological features confer ecological fitness and transmissibility? And how do lineage-specific traits contribute to pathogenesis?

In the United States (U.S.), many studies (Table 1) [6,7,8,9,10,11,12,13,14,15,16,17,18,19,20,21] have demonstrated that since the early 2000s, two lineages of MRSA predominate as causes of human infections: USA300, which belongs to sequence type 8 (ST8) within clonal complex 8 (CC8), and USA100, which belongs to ST5 within CC5. Although often grouped together as MRSA, these two lineages differ in pathogenetic mechanisms in important ways.

In this perspective, we argue that viewing MRSA as a single clinical entity masks important biological distinctions between its dominant epidemic clones. By highlighting key differences between USA300 and USA100, we redefine these strains as distinct epidemic lineages whose biology carries important implications for surveillance, prevention, and therapeutic strategies.

## 2. Epidemiology and Clinical Features: Two Distinct Biogeographies

*S. aureus* is frequently carried asymptomatically as a human commensal bacterium, and an opportunistic pathogen, switching from colonization to infection. Based on the sampling of limited areas, such as the few square centimeters probed by a nasal swab, approximately 20% of the human population have been classified as persistent *S. aureus* carriers, and another 20% are considered to be noncarriers. The remaining 60% of the population belong to the group of intermittent carriers [22]. Although it is a convenient classification system, the reality more likely is that if the entire body were swabbed including the groin and other areas known to commonly be colonized by *S. aureus* [23], the vast majority of humans would likely be found to consistently harbor *S. aureus*, making it formally part of the normal human microbiome. It is likely that ready detection by limited nasal swabbing reflects higher-abundance carriage which has proven to be a useful indicator of infection probability. It is known that MRSA colonization as determined by nasal swabbing is a risk factor for MRSA infections [13,20]. At the same time, however, it is unlikely that nasal-swab *S. aureus*-free individuals are truly free of *S. aureus*. In 50% to more than 80% of individuals, the strains that colonize their bodies genetically match the strains that cause infections [20,24,25].

Beginning as early as 1961, MRSA infections were primarily associated with healthcare contact [26]. However, in the 1990s, cases of MRSA infection emerged in individuals with no previous hospitalization, leading to the establishment of separate definitions for hospital-acquired (HA)-MRSA and community-acquired (CA)-MRSA. By the year 2000, USA300 became the dominant strain causing CA-MRSA infections in the U.S., which increasingly occurred in previously healthy, younger patients and were primarily associated with skin and soft tissue infections (SSTIs) [27]. USA300 has also been linked to severely acute syndromes such as necrotizing pneumonia and severe sepsis [28,29]. USA300 has also been detected outside the U.S., in Europe, South America, and Asia, but has not widely replaced local CA-MRSA strains in these areas [30]. In contrast, HA-MRSA strains are predominantly isolated from individuals exposed to a healthcare setting, who are typically older and have one or more underlying medical conditions [8]. Since the development of the multilocus-sequencing tools that allowed the resolution of genetic lineages of MRSA [31], USA100 is consistently reported among the most prevalent clones causing hospital-associated infections in North America, including bacteremia [13,15,19], pneumonia [7], and invasive infections [8], across wide geographies. Yet the once-clear distinction between CA-MRSA and HA-MRSA infection appears to become increasingly blurred. Carriage rates vary depending on the study conducted, but USA300 has become increasingly prevalent as a cause of asymptomatic colonization in the general population [32]. Some MRSA USA100 have been isolated from infections occurring in non-hospitalized patients, such as being the most common lineage identified in cases of MRSA keratitis [33]. Interestingly, in ophthalmology, differences from the keratinized surfaces of the surrounding adnexa have profound consequences for the types of MRSA that infect wet versus keratinized ocular epithelial tissues. These two clones have strong tropisms for distinct biogeographies, suggesting lineage-specific host adaptation. We found that the USA100 MRSA lineage is predisposed to infect the cornea, whereas the USA300 MRSA lineage selectively infects surrounding skin and soft tissue [18,33]. Furthermore, numerous studies have identified USA300 as the cause of nosocomial MRSA outbreaks and infections among patients with chronic illnesses [6,34]. Taken together, these findings highlight the high prevalence of MRSA infections caused by USA100 and USA300 in both community and hospital environments, and that although they exhibit a predisposition for one or the other, their Venn diagrams overlap.

## 3. Microbiological Characteristics: Two Distinct Antibiotic Resistance Patterns

Although, both USA100 and USA300 are methicillin-resistant by carrying the staphylococcal cassette chromosome (SCC*mec*), the SCC*mec* element—and the resistance it confers to other antibiotics—differs profoundly between these two clones.

DNA sequencing showed that MRSA USA100 strains carry large SCC*mec* elements (~53 kb) classified as type II [35]. SCC*mec*-II contains recombinogenic nucleotide sequences where genes responsible for non-β-lactam resistances often insert [36]. As a result, SCC*mec*-II is more commonly associated with multidrug-resistant (MDR) phenotypes. This multidrug-resistant phenotype aligns with constant exposure to antibiotic pressure in healthcare settings and likely contributes to USA100’s ecological success in that environment. In contrast, MRSA USA300 isolates carry a smaller SCC*mec* cassette, termed type IV (~24 kb), which more rarely includes other antibiotic resistance genes.

Although many studies on the antibiotic susceptibility of a large collection of MRSA have been performed [37,38,39,40,41], only a few include genomic characterization of the strains to subclassify them into groups typified by USA300 or USA100 strains [7,12]. These studies are usually conducted during clinical trials. For example, Mendes et al. [7] compared the susceptibility profiles of selected agents tested against a large collection of MRSA clinical isolates recovered during a phase IV pneumonia clinical trial for linezolid (USA300, *n* = 56; USA100, *n* = 110). In this study, USA300 isolates were found to be susceptible to most antimicrobial agents tested (≥85.7% susceptible), except for erythromycin (1.8% susceptible) and gatifloxacin (32.1% susceptible). In contrast, the multiplicity of resistances was higher for USA100, with all of them being resistant to erythromycin and clindamycin, and only 2.7% being susceptible to gatifloxacin. In another study, Richter et al. [12] assessed the activity of ceftaroline against *S. aureus* isolates collected in the U.S. (USA300, *n* = 1137; USA100, *n* = 377). They found that 94% of USA100 were resistant to clindamycin versus 7.6% of USA300; and 95% of USA100 strains were resistant to levofloxacin versus 49% of USA300.

In both USA100 and USA300, resistance to fluoroquinolones is typically attributable to chromosomal mutations that lead to amino acid substitutions in bacterial topoisomerase II and IV [42]. USA100 typically harbors more fluoroquinolone mutations, which is usually reflected in higher levels of fluoroquinolone resistance [33].

Interestingly, most acquisitions of vancomycin resistance have occurred in USA100 or related strains [43], reflective of the establishment of this lineage in the healthcare environment. The first vancomycin resistant MRSA strain was reported in the US in 2002. That strain acquired a VanA phenotype-conferring operon from an *Enterococcus faecalis* donor [44]. The VanA phenotype is characterized by high-level resistance to vancomycin and teicoplanin and is typically mediated by the transposon Tn1546. The repeated acquisition of vancomycin resistance by USA100, along with its early acquisition of methicillin resistance and resistance to other antibiotics, suggests that this lineage may have genetic predispositions for horizontal acquisition of resistance genes. It was previously observed that the USA100 strains that had independently acquired vancomycin resistance operons from enterococci all lacked a variable chromosomal region that in other lineages includes a bacteriocin [43]. In hospital environments where antibiotics are frequently used, this bacteriocin may be obsolete as competitors are eliminated iatrogenically. It has been suggested that the absence of this bacteriocin may facilitate the intimate contact with other resistant microbes that is required for conjugal plasmid transfer [43]. However, VRSA infection remains rare, and there has been no documented transmission of a VRSA strain from one patient to another to date. This is likely explained by the fact that the expression of resistance is highly costly for the host [45]. In addition, the low dissemination of VRSA has also been attributed to the high instability of certain enterococcal plasmids in MRSA isolates [46].

## 4. Genetic Diversity: Two Distinct Virulence Features

Beyond resistance, MRSA USA100 and USA300 strains differ in virulence features as well. Severe and invasive MRSA USA300 infections in previously healthy patients have been reported in various medical centers [47,48]. These infections are often associated with specific staphylococcal syndromes, including necrotizing fasciitis and necrotizing pneumonia, suggesting the presence of unique virulence genes rare in other strains [28].

DNA sequence analysis has identified many candidate factors in the accessory genome of *S. aureus*, potentially contributing to the emergence and success of USA300 and USA100 strains. These include MGEs such as pathogenicity islands, bacteriophages, chromosomal cassettes, transposons, and plasmids.

The accessory genome of the first USA300 strain sequenced is largely clustered into five large genetic elements (>25 kb) on the chromosome, and on three plasmids [49]. The five genetic elements and two of the three plasmids carry known or predicted virulence factors and resistance determinants that likely contribute to the success of USA300 in transmission, colonization and pathogenesis. Notably, Panton–Valentine leucocidin toxin (PVL) is a virulence factor found in USA300 strains associated with community-acquired infection. PVL is a two-component pore-forming protein encoded by the *lukF-PV* and *lukS-PV* genes located in lysogenic prophage ϕSA2 [49]. PVL induces cell death in polymorphonuclear cells. It is strongly associated with SSTIs and necrotizing pneumonia. Higher mortality and an increased likelihood of sepsis, hemoptysis, and pleural effusion have been documented for cases caused by PVL-positive strains [47].

Another important variable genetic element associated with USA300 strains is the arginine catabolic mobile element (ACME). ACME is a 30.9 kb DNA element that is integrated into the same site on the chromosome as the SCC*mec* element [49], suggesting that its integration and excision may be facilitated by the cassette chromosome recombinase (*ccrA/B*). ACME was originally identified in the skin commensal species, *S. epidermidis*, and is believed to enhance survival in the acidic environment typically found on the skin [50]. In USA300, this genetic island contains a putative oligopeptide permease operon (*Opp-3*) and a group of six genes known as the *arc* gene cluster, which encodes an arginine deiminase pathway that liberates ammonia [49]. In addition to facilitating skin colonization, ACME may also enhance bacterial survival at low pH in intracellular compartments of phagocytic cells, and in abscess cavities. ATP production by the arginine deiminase pathway is induced under anaerobic conditions [51], which could be crucial for energy generation in wound environments with low oxygen levels. Furthermore, since L-arginine is a substrate for nitric oxide production, the depletion of L-arginine by arginine deiminase inhibits the production of nitric oxide, a molecule involved in both innate and adaptive immune responses against microbial infections [52]. In addition, an important mobile genetic element associated with USA300 strains is the pathogenicity island (SaPI) SaPI5 [49], which carries genes encoding superantigen toxins SEQ and SEK [5]. These pyrogenic superantigen toxins stimulate inflammatory cytokine production by leading to antigen-nonspecific activation of the immune system [53]. Although MGEs play an important role in USA300 transmission, colonization and pathogenesis, mutations in core genes also have been identified including those that impact the expression of important virulence factors, such as cytolytic phenol-soluble modulins (PSMs) [54] and α-toxin [55].

Capsular polysaccharides play an important role in the pathogenicity and immunogenicity of *S. aureus* and have been considered as targets for vaccine development [56]. Although both MRSA USA100 and USA300 harbor the cap5 locus, USA300 strains typically do not produce capsule due to conserved mutations in capsule biosynthesis genes, whereas USA100 generally retains functional capsule expression [57,58].

In contrast to USA300, USA100 strains lack PVL. On the other hand, CC5 genomes include a larger set of lipoprotein genes compared to non-CC5 genomes [43]. These lipoproteins modulate host immunity and induce an inflammatory response [59]. Other differences in the distribution of virulence genes in the genomes of USA100 and USA300 *S. aureus* strains are also known. In a study of *S. aureus* blood culture isolates, Smith et al. [15] found that CC5/USA100 typically encoded a much larger repertoire of enterotoxin/superantigen toxins genes compared to that of CC8/USA300. They detected the enterotoxin gene cluster *egc* (*seg*, *sei*, *sem*, *sen*, *seo* and *seu*) in every CC5 genome (*n* = 89) with very few found in CC8 genomes. In contrast, the enterotoxin genes *sek* and *seq* were more common in CC8 (*n* = 48).

Given the crucial role of MRSA virulence factors in disease, they present promising targets for novel therapies, particularly in the face of widespread antimicrobial resistance.

Anti-virulence approaches focus on the inhibition of the *agr* regulatory system [60], transporter blockers [61], and vaccine approaches that target secreted superantigens and toxins [61], monoclonal antibody therapy [62]; however, their potential has yet to be proven effective clinically [61].

## 5. Conclusions

Although USA100 and USA300 were once viewed as predominantly hospital- and community-associated lineages, respectively, both are now established across healthcare and community settings. This highlights the need for ongoing surveillance programs that incorporate genomic characterization, enabling the tracking of lineage dynamics, as well as virulence and resistance evolution, which may influence clinical outcomes and public health interventions. Future research is needed to identify the molecular and evolutionary drivers of MRSA host adaptation, including the role of mobile genetic elements, regulatory networks, and host–pathogen interactions that enable persistence, immune evasion, and transmission across diverse host environments. In particular, comparative genomic and functional studies are needed to elucidate how lineage-specific traits contribute to colonization, infection severity, and niche adaptation. This will provide valuable insights for guiding the design of prevention and targeted therapeutic strategies to treat MRSA infection.

## Figures and Tables

**Table 1:** Prevalence of MRSA lineages causing infections in the U.S. in the last two decades (2004–2024).

Study	Year	State	HA/CAMRSA	Source of infection	MRSA CC %
Seybold et al. [6]	2004	Georgia	HA and CA	BSI	CC8 = 70 CC5 = 30 Others = 15
Mendes et al. [7]	2004–2010	Multicenter study *	HA	Pneumonia	CC5 = 64.6 CC8 = 28.8 CC45 = 2.7 CC30 = 1.5 ST239 = 1.5 Others = 0.7
Limbago et al. [8]	2005–2006	Multicenter study	HA and CA	Invasive infections	CC5 = 55.5 CC8 = 35.3 Others = 9.2
Mendes et al. [9]	2005–2007	Multicenter study *	HA	Pneumonia	CC5 = 64.4 CC8 = 21.4 CC45 = 7.1 CC12 = 7.1
Peterson et al. [10]	2006–2016	Florida	HA and CA	Ocular	CC5 = 51.1 CC8 = 48.9
Hudson et al. [11]	2008–2010	California	HA and CA	BSI, SSTI, Urine	CC8 = 48.6 CC5 = 39.7 Others = 11.7
Richter et al. [12]	2009	Multicenter study	NS	BSI, SSTI, Joint fluid and lower respiratory	CC8 = 51 CC5 = 17 CC1 = 0.5 Others = 31.5
Tenover et al. [13]	2009–2010	Multicenter study	NS	BSI	CC5 = 55.2 CC8 = 37.5 Others = 7.3
Albrecht et al. [14]	2010–2012	Multicenter study	CA	Abscess	CC8 = 98 CC5 = 2
Smith et al. [15]	2010–2018	New Hampshire	HA and CA	BSI	CC8 = 51.9 CC5 = 47.1 CC1 = 1
Souza et al. [16]	2010–2022	New Hampshire	NS	BSI	CC8 = 56.7 CC5 = 31.4 Others = 11.9
Wurster et al. [17]	2014–2017	Massachusetts	HA and CA	Ocular and otolaryngology	CC5 = 56.1 CC8 = 38.5 ST72 = 1.8 CC59 = 1.8 Others = 1.8
André et al. [18]	2014–2021	Massachusetts	HA and CA	Ocular	CC8 = 48.3 CC5 = 41.4 CC59 = 5.7 Others = 4.6
Park et al. [19]	2015	Minnesota	HA and CA	BSI	CC5 = 67 CC8 = 25 Others = 8
Schwarz et al. [20]	2018–2019	New York	HA and CA	BSI	CC8 = 44 CC5 = 41 Others = 15
Hofstetter et al. [21]	2018–2021	Pennsylvania	HA and CA	BSI, SSTI	CC8 = 65.6 CC5 = 27.2 ST72 = 2.2 CC30 = 1.1 Others = 3.9

HA: hospital acquired; CA: community acquired; BSI: bloodstream infection; SSTI: skin and soft tissue infection; NS: not specified. * only data from U.S. isolates were included in this table.

## Data Availability

All data presented in this perspective are derived from and available in the cited references.
